# Osteonecrosis in the both femoral heads in a patient with Neuro-Behcet’s Disease

**DOI:** 10.12669/pjms.333.12815

**Published:** 2017

**Authors:** Asuman Orhan Varoglu, Asude Aksoy

**Affiliations:** 1 Asuman Orhan Varoglu, Department of Neurology, Medical School, Medeniyet University, Istanbul, Turkey; 2 Asude Aksoy, Department of Internal Medicine, Medical School, Firat University, Elazıg, Turkey

**Keywords:** Neuro-Behçet’s Disease (NBD), osteonecrosis, and both femoral heads

## Abstract

Neuro-Behçet’s disease (NBD) is a rare clinical entity that frequently presents neuro-psychiatric symptoms, and mesodiencephalic and pontobulbar lesions. There is only one published report about osteonecrosis in NBD. We report a patient whose first presentation was neurological NBD with presenting bilateral femoral heads osteonecrosis. A 36-year-old male was hospitalized with gait disorder, diplopia and speech disorder. The past medical history of the patient was unremarkable. The MR image showed mesencephalic lesion with oedemaas a hyperintense area. The present case was diagnosed as NBD and treated with methylprednisolone (1g /day) only for five days. One year after, bilateral hip pain developed. MR image of both hips showed well-demarcated areas of osteonecrosis in the bilateral femoral heads. The patient was operated by an orthopedic surgeon. Because early diagnosis and immediate treatment of osteonecrosis is very important, the physician must bear in mind that osteonecrosis might result from impaired microvascular involvement in even NBD.

## INTRODUCTION

Behçet’s disease has a triad of recurrent oral and genital ulcers and uveitis, although the central nervous system (CNS), gastrointestinal tract, joints, blood vessels and lungs may be involved.^1^ A few reports have shown BD presenting as osteonecrosis.^2^-^5^ There is only one published report about osteonecrosis in NBD patient.^5^ Here, we report a patient whose first presentation was neurological NBD with presenting bilateral femoral heads osteonecrosis.

## CASE REPORT

A 36-year-old male patient was admitted to our clinic because of gait disorder, diplopia, and speech disorder. The patient experienced these symptoms for two weeks. The past medical history of the patient was unremarkable, and the family history was negative for neurological disorders. Oral aphthous ulcerations had been presented for approximately three months.

**Fig.1:**
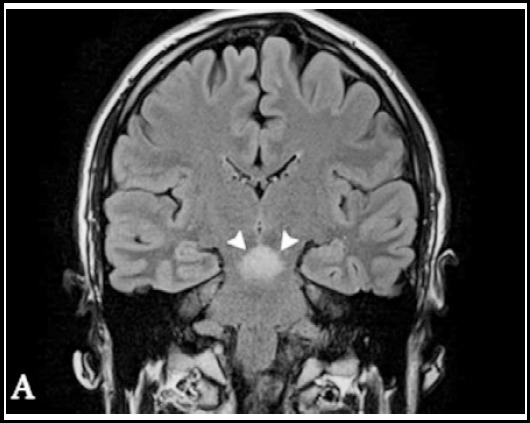
Coronal inversion recovery MR image shows mesencephalic lesion with oedemaas a hyperintense area (arrowheads).

The patient’s neurological examination revealed the presence of cerebellar-type dysarthria, truncal ataxia, wide-based ataxic gait, dysmetria, dysdiadochokinesia, and hyperreflexia in all his extremities. Body temperature was 36,0 °C. MR image showed mesencephalic lesion with oedemaas a hyperintense area (arrowheads) (Fig.1). Contrast uptake was not seen in the MRI. Uveit was not found in the patient’s ophthalmic examination. Human leukocyte antigen B51 (HLADR B1) was found. The markers of collagen tissue disease were negative. Hematological markers, such as fibrinogen, antithrombin, and protein C and S were also in normal ranges, excluding ischemic stroke. Factor V Leiden, prothrombin G20210A gene mutations and MTHFR enzyme were not detected. Transthoracic and transesophageal echocardiography and carotidartery doppler were normal. The plasma Vit B (12), folic acid, and homocysteine levels were normal. Finally, the case was diagnosed as NBD.

At the beginning of therapy, intravenous (IVP) methylprednisolone (1g/day) was administered for five days. The patient’s all neurological deficits improved within two weeks, and the patient was discharged.

**Fig.2:**
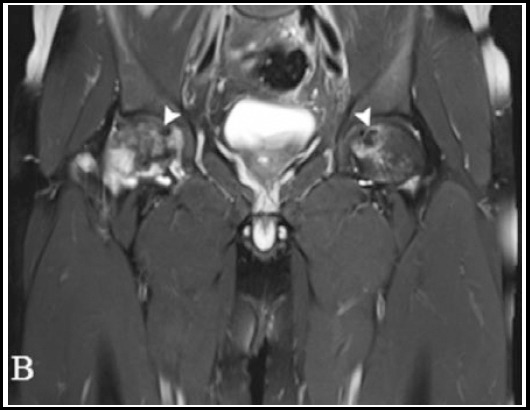
Coronal T2-weighted MR image of both hips shows well-demarcated areas of osteonecrosis (arrowheads) in the both femoral heads and bone marrow oedema in the right femoral heads.

The follow up determined that genital aphthous ulcerations had developed within three months. A relapse of NBD was not seen in this patient, and recurrent corticosteroid and any drugs were not used. However, one year after NBD was diagnosed, the patient consulted an orthopedic surgeon for bilateral hip pain. MR image of both hips showed well-demarcated areas of osteonecrosis (arrowheads) in both femoral heads and bone marrow oedema in the right femoral head (Fig.2). The patient underwent surgery; there were no complications perioperatively, and the patient was discharged.

## DISCUSSION

BS is a chronic, multisystem, inflammatory, and autoimmune disease that affects many organs and systems. The central nervous system’s involvement of BS in patients ranges from 2.2% to 49%.^1^ Neurological involvement in this syndrome is associated with a poor prognosis. The onset of neurological symptoms was reported to be the first manifestation of NBD. Our case was not diagnosed as BS until admission. The first presentation of our case was neurological. In this reported case, characteristic lesions were apparent in MRI. Therefore, our case was diagnosed as NBD.

Primary vascular endothelial cells have been considered to be the original autoimmune target of BD. The cause of oxidative stress increases chronic inflammation and accordingly contributes endothelial cell damage resulting vasculitis.^2^. Therefore, vasculer complications were seen in BD.^3^

A few cases with osteonecrosis in BD have been reported.^2^-^4^ Among these reports, only Chang et al^3^ reported that osteonecrosis of the left knee developed without the use of corticosteroids in BD. Also, depending on the use of corticosteroids, In 1981, Ronco et al.^5^ reported that four patients with Behçet’s disease, who had received high doses of prednisone (0.5 to 1 mg/kg/day) during a mean period of 21 months developed aseptic osteonecrosis of the femoral heads.

In NBD, one case report concerns extensive osteonecrosis at the whole thoracal spinal cord.^6^ This patient had BD for eight years. Paraplegia developed as a result of thoracal spinal cord involvement. Therefore, this patent was diagnosed with NBD. Our case differs from these published case reports because the first presentation was neurological, and there is no symptom as a systemic. In the literature, there is no reported NB patient with osteonecrosis, which has only neurological as the first presentation. The cause of osteonecrosis in both femoral heads might be impaired microvascular involvement resulting from NBD. Therefore, it can be thought that even in the patients, who do not have any systemic signs and symptom and the first presentation is NBD, impaired microvascular involvement is more widespread than expected. Even in the patients who have the first presentation is NBD, systemic sign and symptoms including osteonecrosis may be seen resulting from impaired widespread endothelial dysfunction.

The early diagnosis and immediate treatment of osteonecrosis are very important because of the high potential morbidity. As such physicians should be alert to the presence of systemic sign and symptom for impaired endothelial dysfunction in both BD and even NBD patients.

### Author’s Contribution

**Varoglu AO** conceived, designed and did manuscript writing.

**Aksoy A** did editing of manuscript.
